# Cancer cell–induced neutrophil extracellular traps promote both hypercoagulability and cancer progression

**DOI:** 10.1371/journal.pone.0216055

**Published:** 2019-04-29

**Authors:** Hye Soo Jung, JaYoon Gu, Ji-Eun Kim, Youngwon Nam, Jae Woo Song, Hyun Kyung Kim

**Affiliations:** 1 Department of Laboratory Medicine, Seoul National University College of Medicine, Seoul, Republic of Korea; 2 Cancer Research Institute, Seoul National University College of Medicine, Seoul, Republic of Korea; 3 Department of Surgery, Yonsei University College of Medicine, Seoul, Republic of Korea; 4 Department of Laboratory Medicine, Yonsei University College of Medicine, Seoul, Republic of Korea; University of South Alabama Mitchell Cancer Institute, UNITED STATES

## Abstract

**Introduction:**

Neutrophils can generate extracellular net-like structures by releasing their DNA–histone complexes and antimicrobial peptides, which is called neutrophil extracellular traps (NETs). Various stimuli can induce NET formation. In particular, neutrophils and NET formation are abundant in tumor tissue. This study investigated how cancer cells induce NET formation and whether this NET formation promotes plasma thrombin generation and cancer progression.

**Methods:**

Induction of NET formation by a pancreatic cancer cell line (AsPC-1) was assessed by measuring the histone–DNA complex level. The endogenous thrombin potential (ETP) was measured by thrombin generation assay. *In vitro* migration, invasion, and tubule formation assays were performed. The circulating levels of NET markers and hypercoagulability markers were assessed in 62 patients with pancreatobiliary malignancy and 30 healthy controls.

**Results:**

AsPC-1 significantly induced NET formation in a dose-dependent manner. Conditioned medium (CM) from AsPC-1 also induced NETs. Interestingly, NET-formation was abolished by heat-inactivated CM, but not by lipid-extracted CM, suggesting an important role of protein components. A reactive oxygen species inhibitor did not inhibit cancer cell–induced NET formation, but prostaglandin E1 (PGE1, cyclic adenosine monophosphate inducer) and antithrombin did. NETs significantly increased ETP of normal plasma. Of note, NETs promoted cancer cell migration and invasion as well as angiogenesis, which were inhibited by histone-binding agents (heparin, polysialic acid), a DNA-degrading enzyme, and Toll-like receptor neutralizing antibodies. In patients with pancreatobiliary malignancy, elevated NET markers correlated well with hypercoagulability makers.

**Conclusion:**

Our findings indicate that cancer cell–induced NET formation enhances both hypercoagulability and cancer progression and suggest that inhibitors of NET formation such as PGE1 and antithrombin can be potential therapeutics to reduce both hypercoagulability and cancer progression.

## Introduction

In response to various stimuli such as pathogens and inflammatory cytokines, neutrophils release net-like structures that consist of their DNA–histone complexes and antimicrobial peptides such as neutrophil elastase (NE) and matrix metalloproteinase 9 (MMP9), which is called neutrophil extracellular traps (NETs) [1–3]. Reactive oxygen species (ROS) mediate some forms of NET formation [4]. The NETs play a role in immune protection through killing pathogens, but they can be detrimental in thrombotic and inflammatory diseases [5].

Neutrophils and NETs are abundant in tumor tissue [6]. It has been reported that malignant neutrophils are prone to NET formation and that cancer cells and cancer cell–primed platelets could also induce NET formation [7, 8]. However, it remains to be investigated how cancer cells induce NET formation.

NETs can promote thrombosis in multiple ways [9]. They bind to platelets, activate the coagulation system, and inhibit activation of the anticoagulant system and fibrinolysis [9]. Since neutrophils and NETs are abundant in tumor tissue, the NETs have sparked much interest in tumor-associated thrombosis [1]. In mice, tumor injection induced NET formation and lung thrombosis [10] and NET formation occurred concomitant with thrombosis appearance in tumor-bearing mice [8]. Cancer is often accompanied by hypercoagulability, which is an abnormal state of blood coagulation that increases thrombosis risk [11]. Among hypercoagulability markers, circulating microparticles are considered to be a potent procoagulant and biomarker of thrombosis in cancer [12]. Endogenous thrombin potential (ETP) represents total thrombin amount in human plasma stimulated by tissue factor determined by using thrombin generation assay and is a sensitive marker of hypercoagulability [13, 14]. Until now, it has been unclear how the NETs influence thrombin generation in cancer.

NETs reportedly promote tumor metastasis [1, 15]. NETs are associated with poor prognosis in cancer, and the soluble mediators from NETs such as NE and MMP9 promoted tumor cell growth [1, 16]. However, the detailed mechanism of NET-induced tumor progression including migration and angiogenesis needs to be clarified.

Pancreatic cancer not only shows high metastasis potential [17], but also poses a serious risk of cancer thrombosis [18]. In this study, we hypothesized that pancreatic cancer cells by itself induce NET formation, resulting in both hypercoagulability and tumor progression. We investigated whether how pancreatic cells induced NET formation and whether NETs promoted plasma thrombin generation. We also investigated NET-induced cancer cell migration, invasion, and angiogenesis. Finally, we measured the circulating levels of NET markers (histone–DNA complex, cell-free dsDNA) and hypercoagulability markers (microparticles, ETP) in patients with pancreatobiliary cancer to assess the relationship between NETs and hypercoagulability.

## Materials and methods

### Cell culture

The human pancreatic carcinoma cell line AsPC-1 and human gastric carcinoma cell line NCI-N87 were cultured in RPMI 1640 (WelGENE, Seoul, South Korea) with 10% fetal bovine serum (FBS; Gibco, Grand Island, NY, USA). The human endothelial cell line EA.hy926 and human umbilical vein endothelial cells HUVEC were cultured in DMEM medium (WelGENE) with 10% FBS (Gibco) and EBM-2 medium (Clonetics, San Diego, CA, USA) with EGM-2 Endothelial SingleQuots Kit (Clonetics), respectively.

### Measurement of AsPC-1-induced NET formation and ROS activity

Peripheral whole blood was collected from healthy volunteers under informed consent. Blood samples were incubated with AsPC-1 cells for 30 min, 1 h, or 2 h at 37°C and then the supernatants were collected by centrifugation at 3000 ×*g* for 10 min. The histone–DNA complex levels, which reflect the extent of NET formation, were measured by using a Cell Death Detection ELISA kit (Roche Diagnostics, Indianapolis, IN, USA). The percent coefficient variation of the assay was 10.6%. For investigation of dose dependency, various concentrations (0, 5 × 10^4^, and 20 × 10^4^ cells/mL) of AsPC-1 cells were incubated with blood samples for 2 h at 37°C. Additionally, NCI-N87 cells were used to investigate whether additional type of cancer cell line induces NET formation. Phorbol 12-myristate-13-acetate (PMA, 25 nM; Sigma-Aldrich, St. Louis, MO, USA) was used as a positive control of NET formation. HUVEC were used as a negative normal control of NET formation.

Neutrophils were isolated as described previously [19]. Briefly, after erythrocyte aggregation and sedimentation with hydroxyethyl starch (Pentaspan, Jeil Pharmaceutical, Seoul, Korea), peripheral whole blood was treated with Ficoll-Paque with a density of 1.077 (Sigma-Aldrich) to separate the mononuclear cells from neutrophils. The purity of the isolated neutrophils was confirmed to be >85% of total cells through flow cytometric analysis by using anti-CD33 antibody (Becton Dickinson, Franklin Lakes, NJ, USA).

To prepare conditioned medium (CM), AsPC-1 cells were cultured in RPMI 1640 with 1% FBS (1% FBS RPMI) for 36 h, and the supernatant was obtained by centrifugation at 12,000 ×*g* for 10 min. To eliminate lipid and protein components from CM, lipids were extracted by incubation with 1.5% charcoal (Sigma-Aldrich) at 4°C for 24 h and proteins were degraded by heating CM at 65°C for 1 h.

Inhibitors, namely diphenyleneiodonium (DPI, 20 μM; Tocris Bioscience, Bristol, UK), prostaglandin E1 (PGE1, 1 μg/mL; Mitsubishi Tanabe Pharma Korea, Seoul, Korea), antithrombin (5 IU/mL, SK Plasma, Gyeonggi, Korea), and monoclonal antibody against human tissue factor (anti-TF, 30 μg/mL; clone VD8, American Diagnostica Inc., Stamford, CT, USA) were pre-incubated with peripheral whole blood before adding AsPC-1 cells.

The levels of ROS activity were measured by using an OxiSelect *In Vitro* ROS/RNS assay kit (Cell Biolabs, San Diego, CA, USA).

### Preparation of NETs

Isolated human neutrophils (3.3 × 10^5^ cells/mL) suspended in 1% FBS RPMI were treated with 25 nM PMA for 1 h at 37°C and were washed by centrifugation (1,800 ×*g*, 10 min). NETs were prepared by suspending the pellets with 1% FBS RPMI (3.3 × 10^5^ cells/mL) and protein concentration was adjusted to 300 mg/dL.

### Thrombin generation assay

Thrombin generation assay was performed using commercial normal plasma (Pool Norm; Diagnostica Stago, Asnieres, France) with or without NETs addition according to the previous report [20]. Briefly, 80 μL normal plasma pre-mixed with 20 μL NETs or 1% FBS RPMI (control) was stimulated with 5, 1, or 0.5 pM TF (American Diagnostica). After 20 μL substrate (FluCa-Kit, Thrombinoscope BV, Maastricht, Netherlands) addition, the amounts of generated thrombin were measured using Thrombinoscope software (Thrombinoscope BV).

### Migration and invasion assay

For migration assay, AsPC-1 cells were loaded into the upper chamber of a cell culture insert with 8 μM pore size (Corning Inc., Corning, NY, USA) and the lower chamber was supplemented with intact isolated neutrophils (3.3 × 10^5^ cells/mL) or NETs (300 mg/dL of protein). After 22 h incubation at 37°C, migrated cells were fixed and stained with Diff-Quik kit (Sysmex Co., Kobe, Japan) and were counted under a Leica DM IL LED inverted microscope (Leica Microsystems GmbH, Wetzlar, Germany) using LAS software v.4.0 (Leica Microsystems GmbH) at 400× magnification.

In experiments with NET inhibitors, NETs were pre-treated with heparin (200 IU/mL, Sigma-Aldrich) for 20 min at room temperature, polysialic acid (PSA, 62.5 μg/mL; Sigma-Aldrich) for 1 h at 37°C, or DNase I (50 IU/m, Worthington Biochemical Co., Lakewood, NJ, USA) for 20 min at 37°C. AsPC-1 cells were pre-treated with neutralizing antibodies against Toll-like receptors (TLRs) for 1 h at room temperature by adding anti-TLR2 antibody (aTLR2, 50 μg/mL; eBioscience, San Diego, CA, USA), anti-TLR4 antibody (aTLR4, 50 μg/mL; eBioscience), or a monoclonal mouse IgG_2a,k_ antibody (Iso-IgG, 50 μg/mL; eBioscience) as an isotype control. For invasion assay, a rehydrated Matrigel invasion chamber with 8 μM pore size (Corning Inc.) was used.

### Tubule formation assay

EA.hy926 cells were seeded at 5 × 10^4^ cells per well coated with 200 μL/well of Matrigel (Becton Dickinson Labware, Bedford, MA, USA) and incubated for 4 h at 37°C in DMEM medium with or without calf thymus histones (Roche Diagnostics). The capillary-like structures were then examined under an Olympus CKX41SF microscope (Olympus Co., Tokyo, Japan), and tubule length was calculated in four-randomly selected fields using Image J software (NIH, Bethesda, MD, USA).

### Measurement of NET and hypercoagulability markers in patients with pancreatobiliary malignancy

A total of 62 adult patients with pancreatobiliary malignancy were included in the present study. Pancreaticobiliary malignancy was diagnosed based on clinical, laboratory, radiologic, and pathologic findings. Cancer staging was based on radiologic and pathological findings. Peripheral blood samples were collected in 0.109 mol/L sodium citrate (Becton Dickinson, San Jose, CA, USA). Plasma was separated by centrifugation of whole blood at 1550 ×*g* for 15 min, aliquoted and stored at −80°C. Control plasma from healthy adults (n = 30) was also included. This study was approved by the Seoul National University Hospital Institutional Review Board (approval No. 1711-020-897), and written informed consent was obtained from all subjects.The study was approved by the Seoul National University Hospital Institutional Review Board (approval No. 1711-020-897), and written informed consent was obtained from all subjectsThe study was approved by the Seoul National University Hospital Institutional Review Board (approval No. 1711-020-897), and written informed consent was obtained from all subjectsThe study was approved by the Seoul National University Hospital Institutional Review Board (approval No. 1711-020-897), and written informed consent was obtained from all subjects.

As NET markers, histone–DNA complex and cell-free dsDNA were measured in plasma from patients and control subjects using Cell Death Detection ELISA kit (Roche Diagnostics) and Quant-iT Picogreen dsDNA assay kit (Thermo Fisher Scientific, Waltham, MA, USA). As hypercoagulability markers, microparticles were measured with a Zymuphen MP-Activity kit (Hyphen BioMed, Neuville-sur-Oise, France) and ETP was measured.

### Statistical analysis

All results are shown as mean ± standard error of the mean. The data were combined data from 3 or more different experiments. All statistical analysis was performed IBM SPSS Statistics version 22.0 for Windows (SPSS Inc., Chicago, IL, USA). For an experiment involving the incubation time course, two-way analysis of variance (ANOVA) followed by Bonferroni’s post-hoc test was used to analyze statistical difference between control and AsPC-1 group over incubation time. For statistical comparison of more than two groups, one-way ANOVA was conducted using Bonferroni’s post-hoc test. The unpaired *t*-tests were used to compare only two groups. *P* values of < 0.05 were considered statistically significant.

## Results

### Pancreatic cancer cells induce NET formation

**Fig 1:**
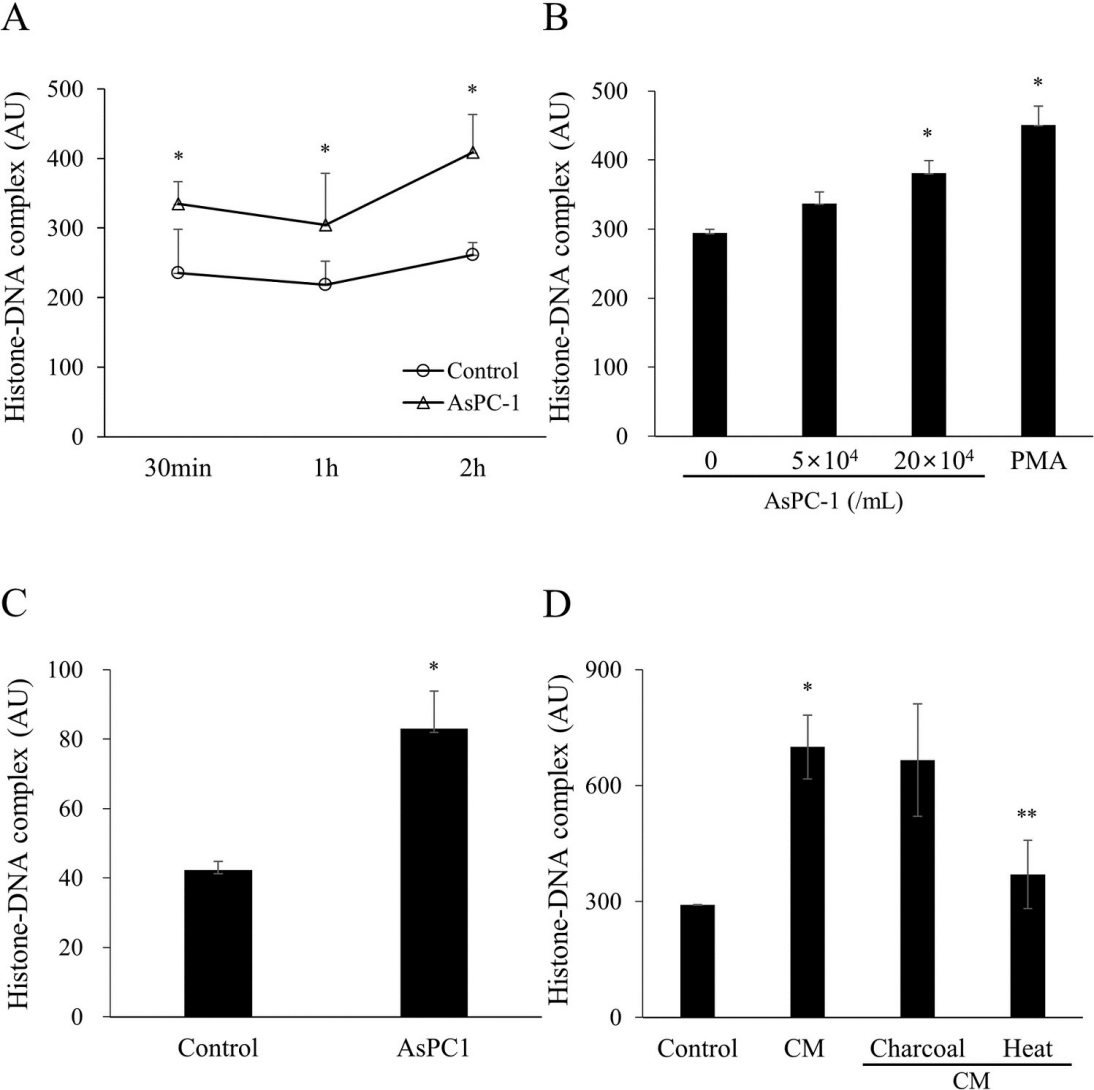
Pancreatic cancer cells AsPC-1 induce neutrophil extracellular traps. (A) AsPC-1 (5 × 10^4^ cells/mL) were incubated with whole blood for 30 min, 1 h, or 2 h at 37°C. Control represents whole blood without AsPC-1. The histone–DNA complex levels were measured in the supernatants. A significant difference was observed among the groups in histone-DNA complex level (NET formation) during incubation time. Statistical analysis was performed by two-way ANOVA followed by Bonferroni’s post-hoc test. ^*^P < 0.05 versus control group. (B) AsPC-1 (5 × 10^4^ or 20 × 10^4^ cells/mL) were incubated with whole blood for 2 h. Phorbol 12-myristate-13-acetate (PMA, 25 nM) was used as a positive control of NET formation. Statistical analysis was performed by one-way ANOVA followed by Bonferroni’s post-hoc test. *P < 0.05 versus whole blood without AsPC-1. (C) AsPC-1 cells were incubated with isolated neutrophils for 2 h and the histone–DNA complex level was measured in the supernatants. (D) Conditioned medium (CM) harvested from AsPC-1 culture was incubated with whole blood for 2h and the histone-DNA complex level was measured in the supernatants. Charcoal was added to CM to remove lipids and heat treatment was used to degrade proteins. Data are expressed as mean ± SEM of 3 experiments. ^*^P < 0.05 versus control; **P < 0.05 versus CM. Abbreviations: AU, arbitrary units.

The histone–DNA complex level, a known NET formation marker [21], in the supernatants of whole blood showed an overall increase in AsPC-1 group compared to control group during incubation time. The results of two-way ANOVA (group × time) in NET formation during incubation time observed no significant interaction (*P* = 0.813) or main effect of time (*P* = 0.305), but a significant main effect of group (*P* = 0.012) (Fig 1A). AsPC-1 increased the histone–DNA complex level in a dose-dependent manner. An increase was also observed after treatment with PMA, a known NET inducer (Fig 1B) [7]. To investigate whether additional type of cancer cell line shows the same effect of NET formation, gastric cancer cells (NCI-N87) were used as well. As expected, NCI-N87 significantly induced NET formation (Figure A in S1 Fig). As a negative control, human umbilical vein endothelial cells (HUVEC) were used. HUVEC did not induce the histone-DNA complex level (Figure B in S1 Fig).

To exclude the effect of other blood cells, we incubated AsPC-1 cells with isolated neutrophils. In this setting, AsPC-1 cells also significantly increased the histone–DNA complex level (Fig 1C).

To investigate whether a direct contact with AsPC-1 cells or soluble factors released by AsPC-1 induce NET formation, CM harvested from AsPC-1 culture was incubated with whole blood. CM increased the histone–DNA complex level (Fig 1D). When lipid components were removed from CM by charcoal treatment, CM still increased the histone–DNA complex level. However, heat-treated CM, in which protein components were degraded, significantly decreased the histone–DNA complex level in comparison with untreated CM (Fig 1D).

### Pancreatic cancer–induced NET formation is not ROS-dependent but cyclic AMP and thrombin-dependent

**Fig 2:**
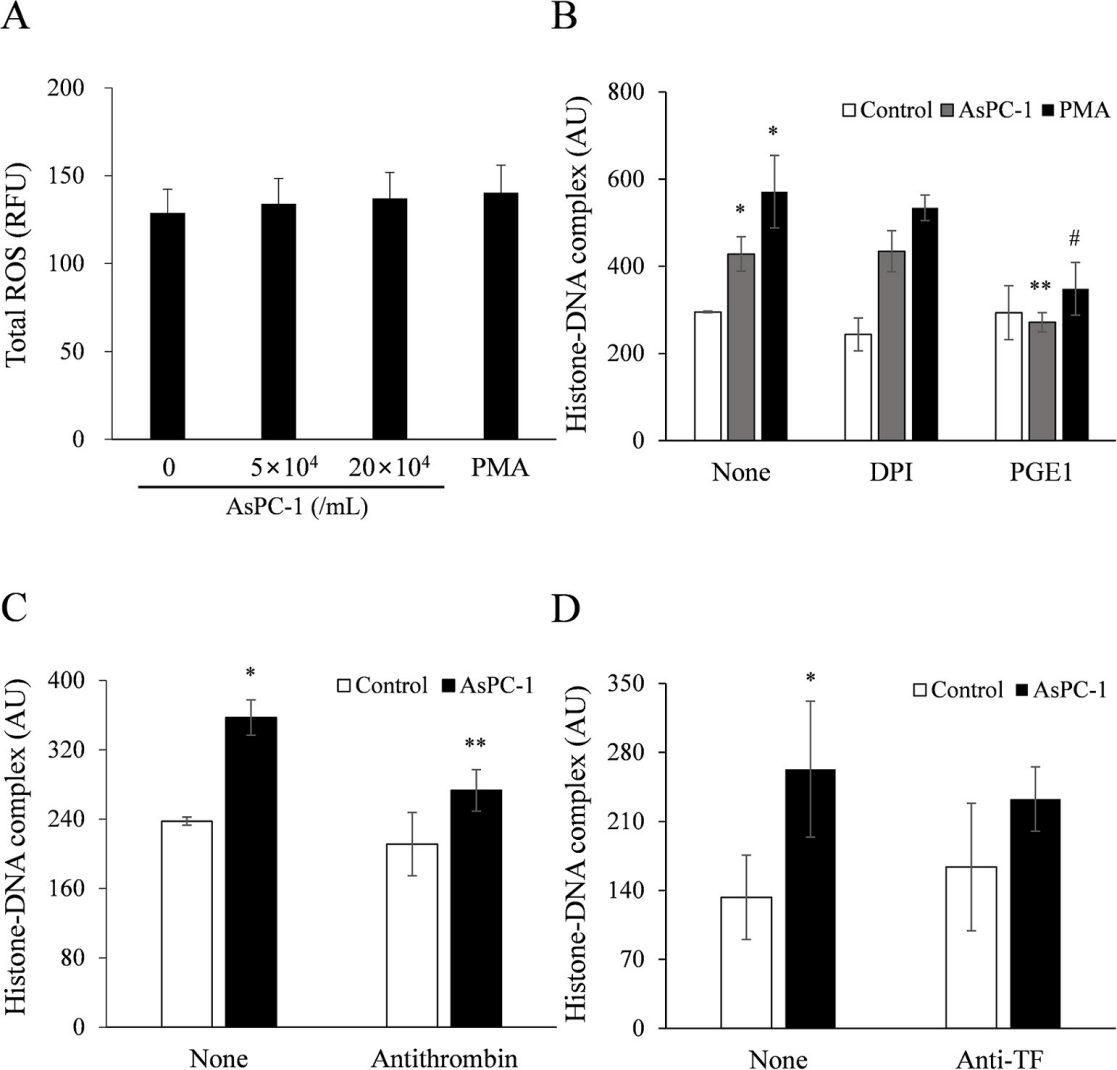
AsPC-1–induced NET formation is not ROS-dependent but cyclic AMP and thrombin-dependent. (A) AsPC-1 or PMA (positive control) was incubated with whole blood for 2 h at 37°C, and total ROS activity was measured in the supernatants. (B) Whole blood was pretreated with diphenyleneiodonium (DPI, 20 μM; ROS inhibitor) or prostaglandin E1 (PGE1, 1 μg/mL; cyclic AMP inducer) for 10 min at room temperature and then incubated with PMA or AsPC-1 for 2 h at 37°C. The histone–DNA complex level was measured in the supernatants. *P < 0.05 versus whole blood without AsPC-1 or PMA-treatment (control); ^**^P < 0.05 versus whole blood with AsPC-1-treatment DPI or PGE1 untreated; ^*#*^*P* < 0.1 versus whole blood with PMA-treatment DPI or PGE1 untreated. (C, D) Whole blood was treated with (C) antithrombin (5 IU/mL) or (D) monoclonal antibody against human tissue factor (anti-TF, 30 μg/mL) for 10 min at 37°C and the histone–DNA complex level was measured in the supernatants. Data are expressed as mean ± SEM of 3 experiments. *P < 0.05 versus whole blood without AsPC-1-treatment (control); ^**^P < 0.05 versus whole blood with AsPC-1-treatment antithrombin untreated. Abbreviations: RFU, relative fluorescence units; AU, arbitrary units.

We investigated whether AsPC-1–induced NETs increase ROS activity in a dose-dependent manner. The ROS activity was not increased in supernatants of whole blood incubated with AsPC-1 cells or PMA (Fig 2A). The ROS inhibitor DPI did not inhibit AsPC-1-induced NET formation (Fig 2B). Interestingly, PGE1, which induces intracellular cyclic AMP (cAMP) production, significantly inhibited AsPC-1-induced NET formation (Fig 2B).

Since thrombin is essential for coagulation and enhances platelet-neutrophil interaction [10], we investigated the effect of a thrombin inhibitor, antithrombin, on AsPC-1-induced NET formation. As expected, antithrombin significantly inhibited AsPC-1-induced NET formation (Fig 2C). Because AsPC-1 cells express surface TF, which can activate the coagulation system, resulting in thrombin production [22], we investigated the effect of a TF inhibitor, the TF neutralizing antibody, but found that it failed to block AsPC-1-induced NET formation (Fig 2D).

### NETs promote thrombin generation

**Fig 3:**
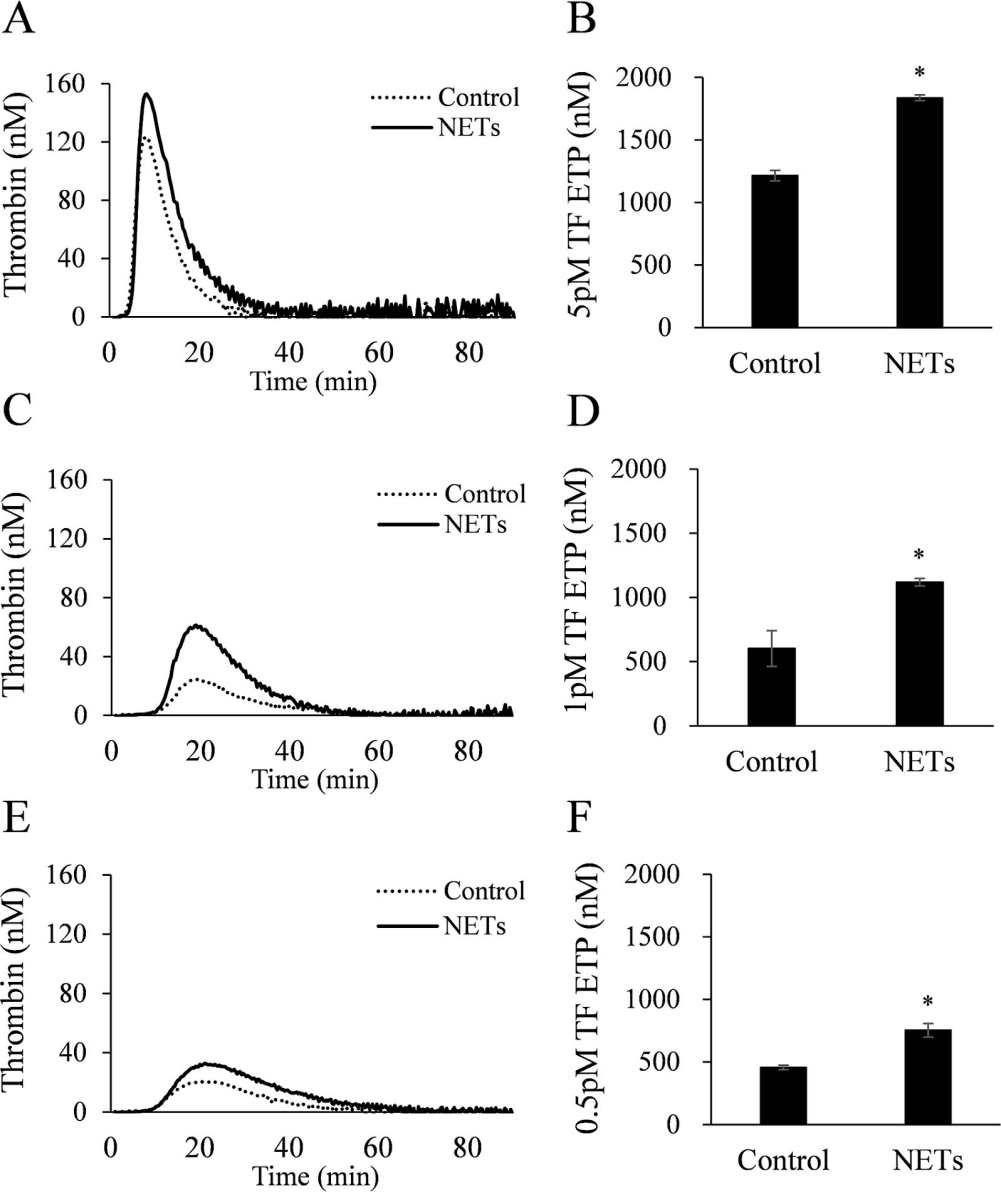
NETs increase thrombin generation in normal plasma. NETs were prepared from isolated neutrophils treated with PMA and added (6.4 mg/dL final protein level) to normal plasma. Endogenous thrombin potential (ETP) was measured by thrombin generation assay after stimulation with (5, 1, or 0.5 pM TF. All data are presented as mean ± SEM from 4 different experiments. ^*^P < 0.05 versus control.

We examined whether NETs increase thrombin generation in normal plasma by using thrombin generation assay. NETs significantly increased ETP levels at 3 different TF concentrations: 5 pM (Fig 3A and 3B), 1 pM (Fig 3C and 3D), and 0.5 pM (Fig 3E and 3F).

### NETs promote cancer cell migration and invasion

**Fig 4:**
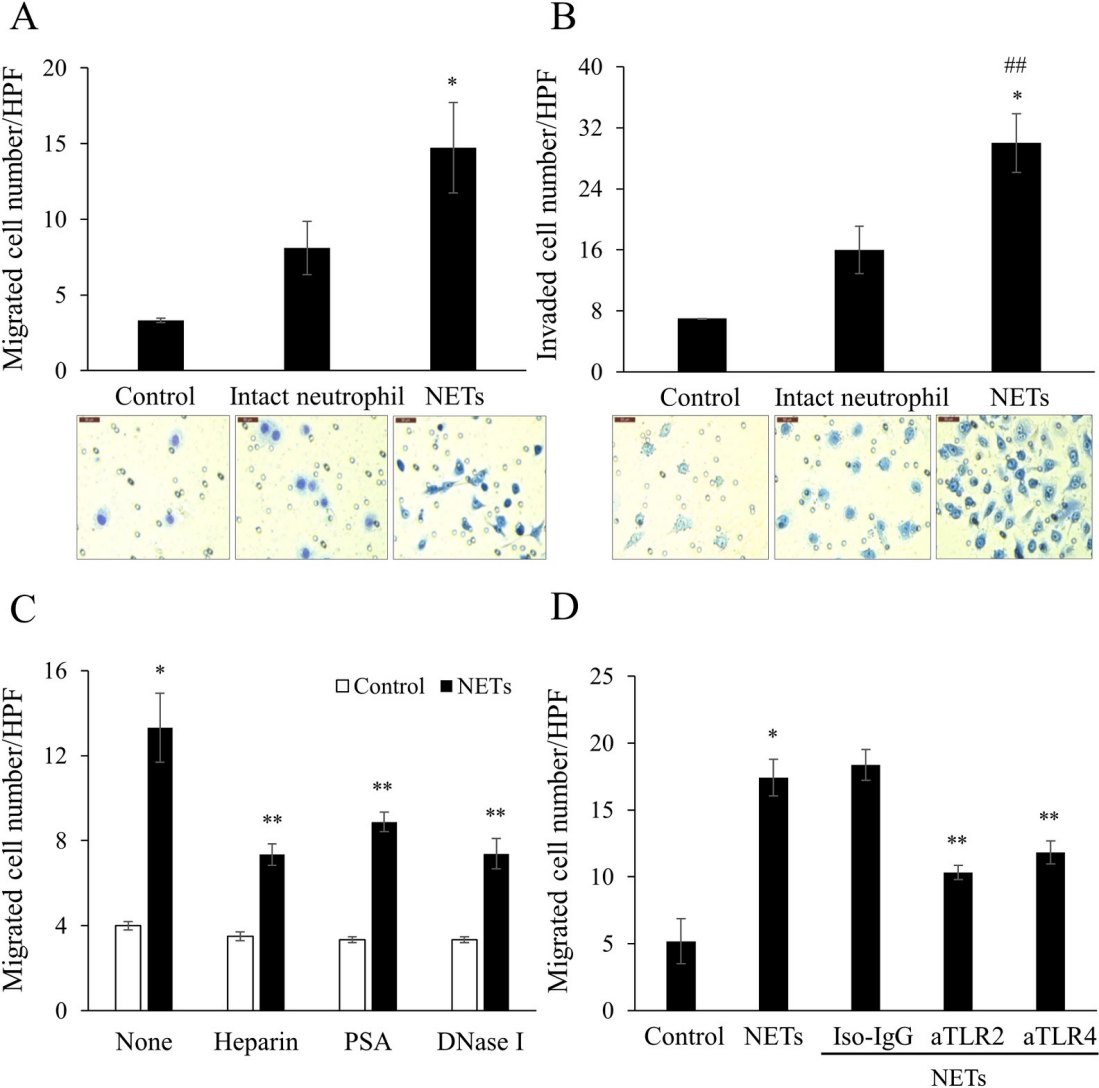
NETs promote migration and invasion of pancreatic cancer cells. Intact isolated neutrophils (3.3 × 10^5^ cells/mL) and NETs (300 mg/dL final protein concentration) were used as chemoattractants. They were added to the lower chambers of (A) an uncoated transwell and (B) rehydrated Matrigel–coated transwell. AsPC-1 (2 × 10^5^ cells/mL) were added to upper chambers. After 22 h incubation, the (A) migrated and (B) invaded cells were stained with Diff-Quik kit and imaged under an optical microscope (×400). Averaged data of six randomly selected high-power fields (HPF) are shown. Scale bar: 50 μm. Statistical analysis was performed by one-way ANOVA followed by Bonferroni’s post-hoc test. ^*^P < 0.05 versus control; ^*##*^*P* < 0.05 versus intact neutrophil. (C) NETs were pre-treated with heparin (200 IU/mL), polysialic acid (PSA, 62.5 μg/mL), or DNase I (50 IU/mL) and then the migration assay was performed. (D) AsPC-1 cells were pre-treated with mouse IgG_2a,K_ antibody (Iso-IgG, 50 μg/mL), anti-Toll-like receptor-2 (aTLR2, 50 μg/mL), or anti-TLR4 (aTLR4, 50 μg/mL) and then the migration assay was performed. The numbers of migrated or invaded AsPC-1 cells are shown as mean ± SEM of 3 experiments. ^*^P < 0.05 versus control; ^*#*^*P* < 0.1 versus control; ^**^P < 0.05 versus NETs.

NETs significantly promoted the AsPC-1 cell migration through a cell culture insert in comparison with vehicle control and intact neutrophils (Fig 4A). Even intact neutrophils promoted AsPC-1 cell migration, although fewer cell migrated than with NETs. NETs also promoted AsPC-1 cell invasion through a rehydrated Matrigel-coated invasion chamber (Fig 4B).

To investigated whether the promotion of cancer cell migration by NETs was specific, we inhibited NETs with histone-binding agents (heparin, PSA) or a DNA cleavage enzyme (DNase I). All 3 inhibitors significantly blocked NET-promoted cancer cell migration (Fig 4C).

Since TLR2 and TLR4 are involved in cell migration and invasion [23, 24], we investigated whether neutralizing antibodies against these receptors (TLR2 and TLR4) would block AsPC-1 cell migration. Both aTLR2 and aTLR4 significantly inhibited NET-promoted AsPC-1 cell migration (Fig 4D), suggesting the involvement of TLR2 and TLR4.

### NETs promote angiogenesis

**Fig 5:**
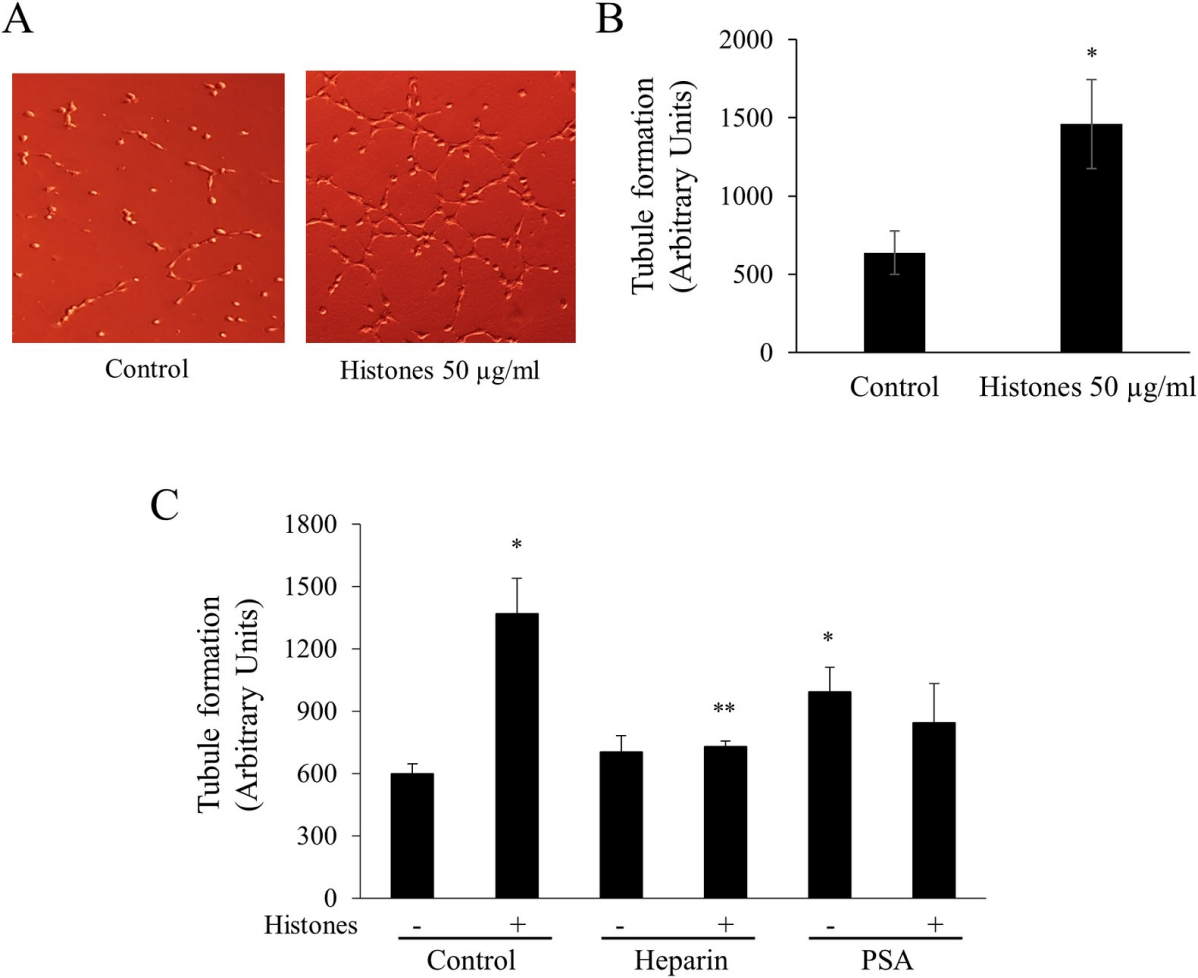
NETs promote endothelial cell angiogenesis. (A) Endothelial cells EA.hy926 were incubated with or without histones (50 μg/mL) for 4 h in a Matrigel–coated well and tubule images were taken under an optical microscope (×100). (B) EA.hy926 (5 × 10^4^ cells/well) were incubated with various concentrations of histones (none, 10, 20, 50, 100 μg/mL) for 4 h in Matrigel-coated wells. The tubule formation length was calculated by Image J software in four randomly selected fields. (C) As inhibitors of tubule formation, heparin (100 IU/mL) and PSA (62.5 μg/mL) were pre-incubated with or without histones for 1 h. EA.hy926 cells were then added for 4 h. *P < 0.05 versus control; ^**^P < 0.05 versus histone-treated.

Since histones are major components of NETs, we examined whether the histones promote *in vitro* angiogenesis. Histones significantly increased the endothelial cell tubule formation in a dose-dependent manner (Fig 5A and 5B and S2 Fig). To confirm the specificity of this effect, we pre-treated histones with histone-binding agents (heparin or PSA) and then incubated with endothelial cells. Both heparin and PSA blocked histone-induced tubule formation (Fig 5C).

### NET and hypercoagulability markers are increased in patients with pancreatobiliary malignancy

**Fig 6:**
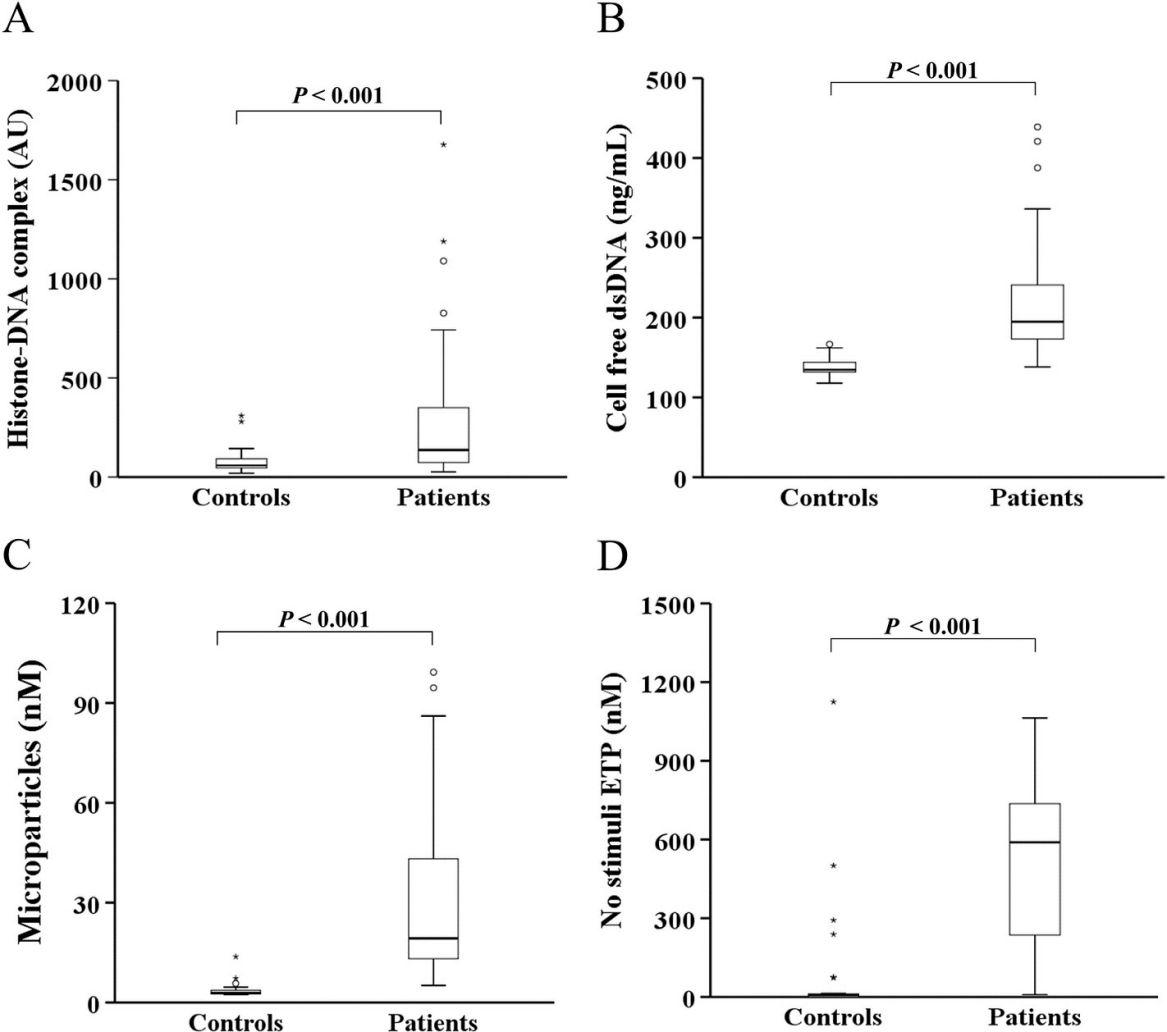
The circulating levels of NET and hypercoagulability markers are increased in patients with pancreatobiliary malignancy. The levels of (A) histone–DNA complex, (B) cell-free dsDNA, (C) microparticles, and (D) ETP in the absence of stimuli (no stimuli ETP) were measured in patients with pancreatobiliary malignancy (n = 62) and healthy controls (n = 30). Abbreviations: AU, arbitrary units.

Since cancer cells promoted NET formation, we examined whether NET formation is increased in cancer patients. As expected, in patients with pancreatobiliary malignancy, the circulating levels of NET markers (histone–DNA complex and cell-free dsDNA) were significantly higher than in healthy controls (Fig 6A and 6B). Similarly, the circulating levels of hypercoagulability markers (microparticles and ETP) were also increased in patients with pancreatobiliary malignancy (Fig 6C and 6D). Interestingly, the microparticle level significantly correlated with histone–DNA complex (*r* = 0.546; *P*<0.01) and cell-free dsDNA levels (*r* = 0.664; *P*<0.01) and so did ETP (*r* = 0.263, *r* = 0.530, respectively; both *P*<0.05).

When all patients with pancreatobiliary malignancy (n = 62) were subdivided into 3 groups based on cancer stage, patients with stage IV (n = 34) tended to show higher levels of NET and hypercoagulability markers than patients with cancer stages I/II (n = 8) and III (n = 20) (S3 Fig).

## Discussion

Our study demonstrated that pancreatic cancer cells induced NET formation that was inhibited by PGE1 or antithrombin. The NETs increased not only hypercoagulability through promotion of plasma thrombin generation, but also cancer progression through promotion of cancer cell migration and invasion and endothelial cell angiogenesis. Both circulating NET and hypercoagulability markers were significantly increased in patients with pancreatobiliary malignancy and NET markers correlated well with hypercoagulability markers.

Our result that pancreatic cancer cells induce NET formation in whole blood in a dose-dependent manner reinforces the recently reported evidence of cancer-induced NET formation *in vivo* and *in vitro* [7, 25]. To determine whether NET formation was induced by blood cells or cell–cell contacts, we cultured cancer cells with isolated neutrophils. Active formation of NETs under these conditions suggests that other blood cells are not necessary for NET formation. Interestingly, cell-free CM harvested from cancer cells also induced NET formation, whereas heat treatment of CM abolished this induction, suggesting a contact-independent role of soluble proteins. Considering that cancer cells can shed microvesicles called exosomes, protein components of cancer cell–derived exosomes are likely to be one of the NET inducers [26].

Since neutrophils can produce ROS that induce NET formation [27], we investigated the role of ROS in NET formation. The ROS activity was not significantly increased, and a ROS inhibitor also did not inhibit NET formation, suggesting that ROS are not involved in pancreatic cancer–induced NET formation. This is consistent with a previous report in which NET induction by pancreatic cancer did not depend on ROS generation [7].

Interestingly, PGE1 inhibited both cancer cell- and PMA-induced NET formation. PGE1 has various pharmacological activities such as anti-platelet, anti-inflammatory and vasodilating effects [28, 29]. PGE1 can induce intracellular cAMP that inhibits NET formation through reduction of intracellular calcium ions [30]. Therefore, cancer cell–induced NET formation is likely to occur through cAMP production.

In our study, antithrombin significantly reduced the cancer cell-induced NET formation. Antithrombin has not only anticoagulant activity, but also anti-inflammatory activity [16]. As an anticoagulant, antithrombin binds to thrombin, quenching the activity of thrombin, which is the central protease in the coagulation system [31]. Antithrombin also binds to syndecan-4 on neutrophils, thus reducing CXCL-2 expression and finally attenuating neutrophil migration to the sites of inflammation [32]. In our *in vitro* system, it is hard to differentiate which effects of antithrombin are responsible for the inhibition of cancer cell–induced NET formation. There has been an interesting study showing attenuation of endotoxemia through inhibition of NET formation by antithrombin in septic mice [16]. Considering that this beneficial effect of antithrombin is helpful for sepsis treatment, the inhibitory effect of antithrombin on cancer cell–induced NET formation may warrant further study for its future clinical application to cancer therapy.

TF is a surface receptor on AsPC-1 cells that can initiate the coagulation system, leading to thrombin [22]. The failure of the TF neutralizing antibody to inhibit cancer cell–induced NET formation suggests that the surface TF of cancer cells does not play a role in NET formation, at least under our *in vitro* conditions.

NETs are mainly composed of cell-free DNA and histones. Since DNA is negatively charged, it can activate the intrinsic coagulation pathway [33]. Hence, we speculate that the DNA of NETs may activate this pathway, eventually leading to thrombin production. In our experiments, thrombin generation in normal plasma was enhanced by NETs. Since thrombin is an essential protease in the coagulation system, enhancement of NET-induced thrombin generation suggests a significant contribution of NETs to hypercoagulability and thrombotic tendency in cancer.

It has been reported that NETs induce cancer metastasis by sequestering circulating tumor cells [15]. Our results showed that NETs increased cancer cell migration and invasion and this effect was specific because the inhibitors of NETs blocked the migration. More interestingly, neutralizing antibodies of TLR2 and TLR4 significantly blocked NET-induced cancer cell migration, suggesting the involvement of TLR2 and TLR4. Although previous reports showed that TLR2 and TLR4 are involved in cell migration and invasion [23, 24], there has been no data about the ligands of cancer cells for TLR2 and TLR4. We showed that NETs as such act as a chemoattractant. According to recent reports [34–36], high mobility group box 1 (HMGB1) is released from NETs and is a ligand for TLR4. Hence, it is assumed that HMGB1-mediated TLR4 signaling is involved in NET-induced cancer cell migration.

Angiogenesis is a complex morphogenetic process, in which resting endothelium acquires a migratory phenotype, migrating through the basement membrane where endothelial cells proliferate and form endothelial sprouts [37]. Angiogenesis is essential for cancer progression [38]. To the best of our knowledge, there has been no report on the effect of NETs on angiogenesis. We showed that a major component of NETs, histones, promoted angiogenesis. This finding reinforces the role of NETs in cancer progression.

Among NET inhibitors, heparin and PSA are anionic substances which bind to positively charged histones and neutralize the histone activity [5, 39], and DNase I degrades the DNA [40]. The NET inhibitors significantly blocked NET-induced cancer cell migration and angiogenesis, suggesting that migration and angiogenesis are specifically promoted by NET. These findings warrant future exploration of the NET inhibitors as therapeutic agents against cancer progression.

Circulating levels of NET markers are increased in various cancers [41, 42]. Although pancreatic cancer–induced NET may have contributed to thrombosis in one *in vitro* study [7], so far there has been no data on circulating NET levels in patients with pancreatic cancer. Our data show that circulating levels of the histone–DNA complex and cell-free dsDNA, which have been proposed as NET markers [21], are significantly increased in patients with pancreatobiliary malignancy. Furthermore, circulating levels of hypercoagulability markers (microparticles and ETP) are also significantly increased in these patients and the NET markers correlated well with hypercoagulability markers, suggesting that NET-induced hypercoagulability occurs in pancreatobiliary malignancy.

This study has several limitations. First, we could not identify the exact chemotactic factor(s) for cancer cell migration. However, HMGB1 can a candidate chemotactic factor, because it is released from NETs and acts as a chemoattractant for cancer cells. Future study is necessary to determine which chemotactic factors in NETs participate in cancer cell migration. Second, we could not demonstrate the direct association of NET and hypercoagulability markers with clinical thrombotic events, because the specimens were collected retrospectively and some of the patients had old thrombotic histories that did not show any association of the markers with thrombosis.

In summary, this study demonstrated that pancreatic cancer cells induced NET formation in contact- and ROS-independent manners. Moreover, cancer cell–induced NET formation was inhibited by PGE1 and antithrombin. NETs could promote not only hypercoagulability through the enhancement of plasma thrombin generation, but also cancer progression through the enhancement of cancer cell migration and angiogenesis (S4 Fig) In patients with pancreatobiliary malignancy, the elevated NET markers correlated well with the hypercoagulability makers. Taken together, these findings indicate that cancer cell–induced NET formation plays an important role in enhancing both hypercoagulability and cancer progression and suggest that inhibitors of NET formation such as PGE1 and antithrombin can be potential therapeutics to reduce both hypercoagulability and cancer progression.

## Supporting information

S1 Fig Neutrophil extracellular traps (NETs) formation by additional type of cancer cell and normal endothelial cell. (PDF)

S2 Fig The black and white image that NETs induce endothelial cells EA.hy926 angiogenesis. (PDF)

S3 Fig Circulating levels of NET and hypercoagulability markers tend to increase with the stage of pancreatobiliary malignancy. (PDF)

S4 Fig Potential mechanism of how cancer cell–induced NET formation promotes both hypercoagulability and cancer progression. (PDF)
