# Supplementary material for: Cancer cell–induced neutrophil extracellular traps promote both hypercoagulability and cancer progression
Source: PLoS One. 2019 Apr 29;14(4):e0216055. doi: 10.1371/journal.pone.0216055 (PMC6488070; doi:10.1371/journal.pone.0216055)
Supplement: S1 Fig — (PDF) [file pone.0216055.s001.pdf]

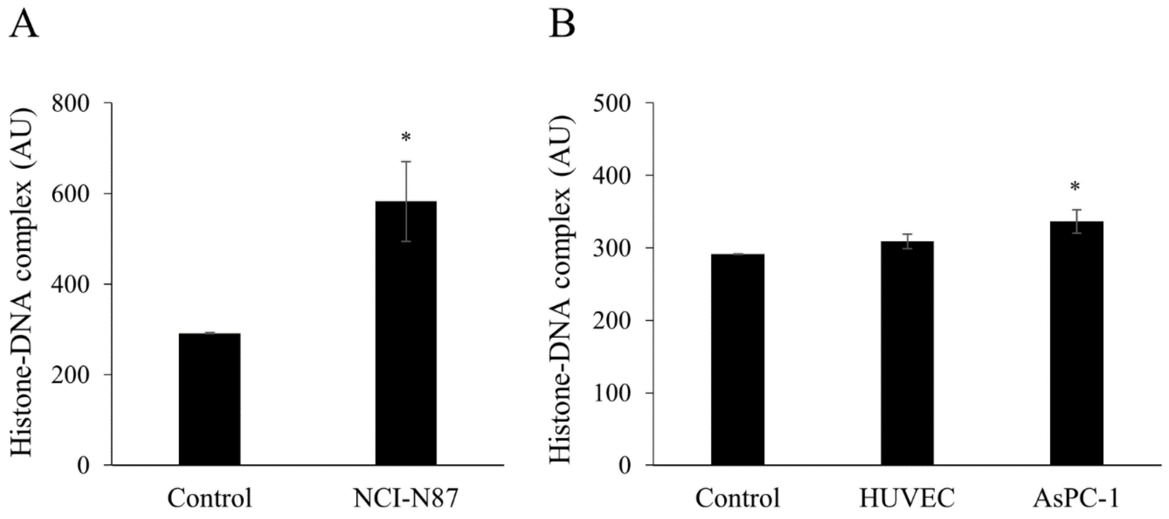

S1 Fig. Neutrophil extracellular traps (NETs) formation by additional type of cancer cell and normal endothelial cell. (A) Gastric cancer cells (NCI-N87, 5×10^4 cells/mL) were incubated with whole bloods for 2 h at 37°C and the histone–DNA complex level was measured in the supernatants. NCI-N87 significantly increased the histone-DNA complex level, suggesting induction of NET formation. (B) Human umbilical endothelial cells (HUVEC) were used as a negative control of NET formation. Whole bloods were incubated with HUVEC (5×10^4 cells/mL) or pancreatic cancer cell line (AsPC-1, 5×10^4 cells/mL) for 2 h at 37°C and the histone–DNA complex level was measured in the supernatants. As expected, HUVEC did not induce NET formation but AsPC-1 significantly induced NET formation. Data are expressed as mean ± SEM of 4 experiments. $^{*}P < 0.05$ versus control (vehicle alone). Abbreviations: AU, arbitrary units.
