# Supplementary material for: Cancer cell–induced neutrophil extracellular traps promote both hypercoagulability and cancer progression
Source: PLoS One. 2019 Apr 29;14(4):e0216055. doi: 10.1371/journal.pone.0216055 (PMC6488070; doi:10.1371/journal.pone.0216055)
Supplement: S2 Fig — (PDF) [file pone.0216055.s002.pdf]

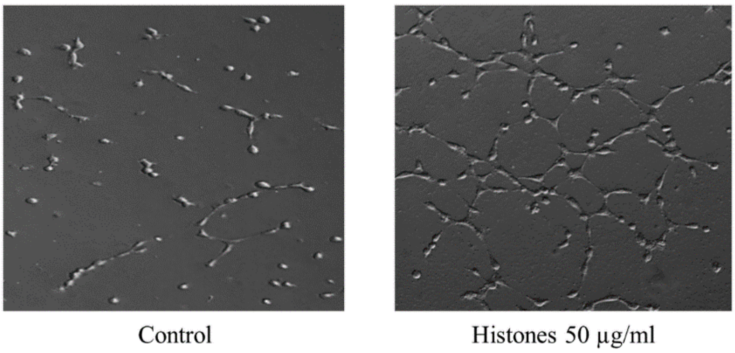

S2 Fig. The black and white image that NETs induce endothelial cells EA.hy926 angiogenesis. Endothelial cells EA.hy926 were incubated with or without histones (50 μg/mL) for 4 h in a Matrigel–coated well and then histones significantly increased the endothelial tubule formation. The capillary-like structures were taken under an optical microscope (×100).
