# Supplementary material for: Cancer cell–induced neutrophil extracellular traps promote both hypercoagulability and cancer progression
Source: PLoS One. 2019 Apr 29;14(4):e0216055. doi: 10.1371/journal.pone.0216055 (PMC6488070; doi:10.1371/journal.pone.0216055)
Supplement: S3 Fig — (PDF) [file pone.0216055.s003.pdf]

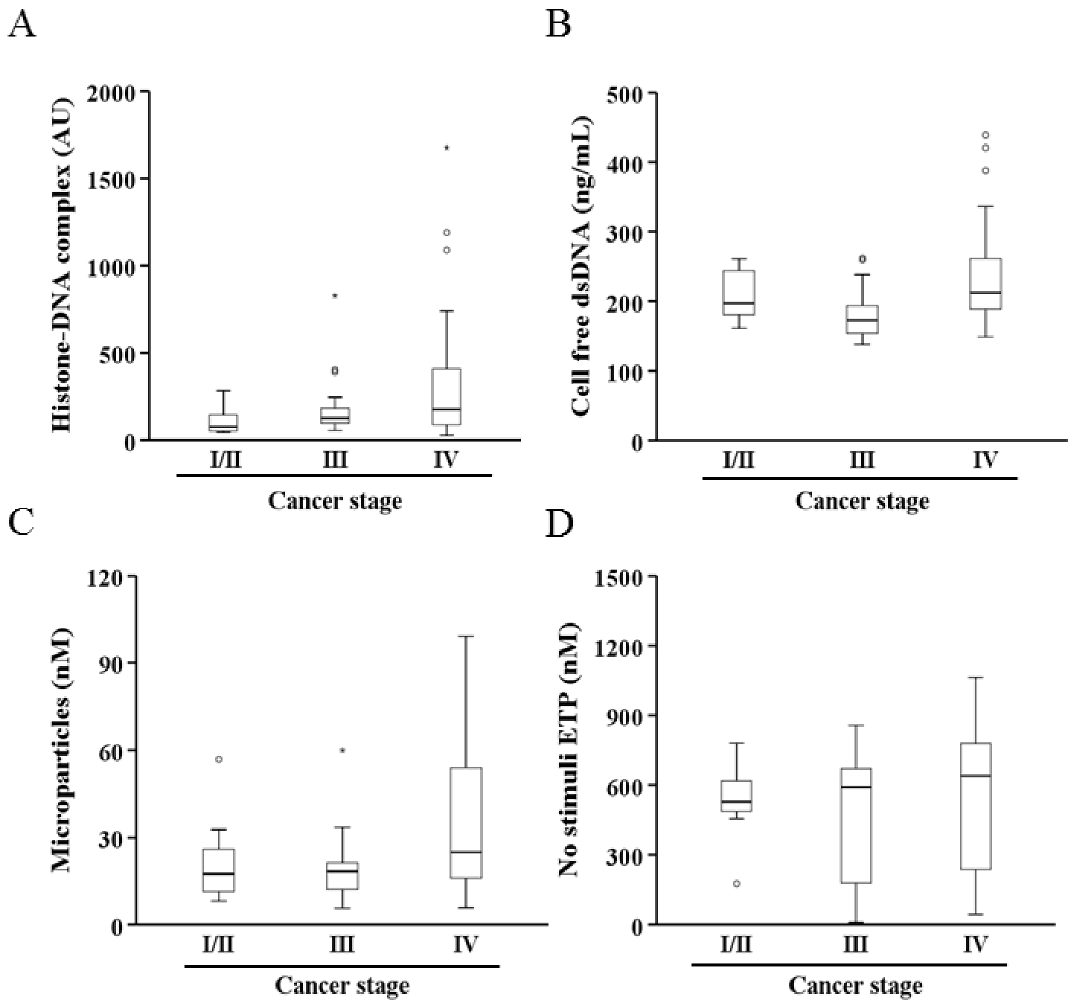

S3 Fig. Circulating levels of NET and hypercoagulability markers tend to increase with the stage of pancreatobiliary malignancy. The levels of (A) histone–DNA complex, (B) cell-free dsDNA, (C) microparticles and (D) ETP in the absence of tissue factor (no stimuli ETP) are shown for patients with stage I/II (n = 8), stage III (n = 20), and stage IV (n = 34) pancreatobiliary malignancy.
