# Supplementary material for: Cancer cell–induced neutrophil extracellular traps promote both hypercoagulability and cancer progression
Source: PLoS One. 2019 Apr 29;14(4):e0216055. doi: 10.1371/journal.pone.0216055 (PMC6488070; doi:10.1371/journal.pone.0216055)
Supplement: S4 Fig — (PDF) [file pone.0216055.s004.pdf]

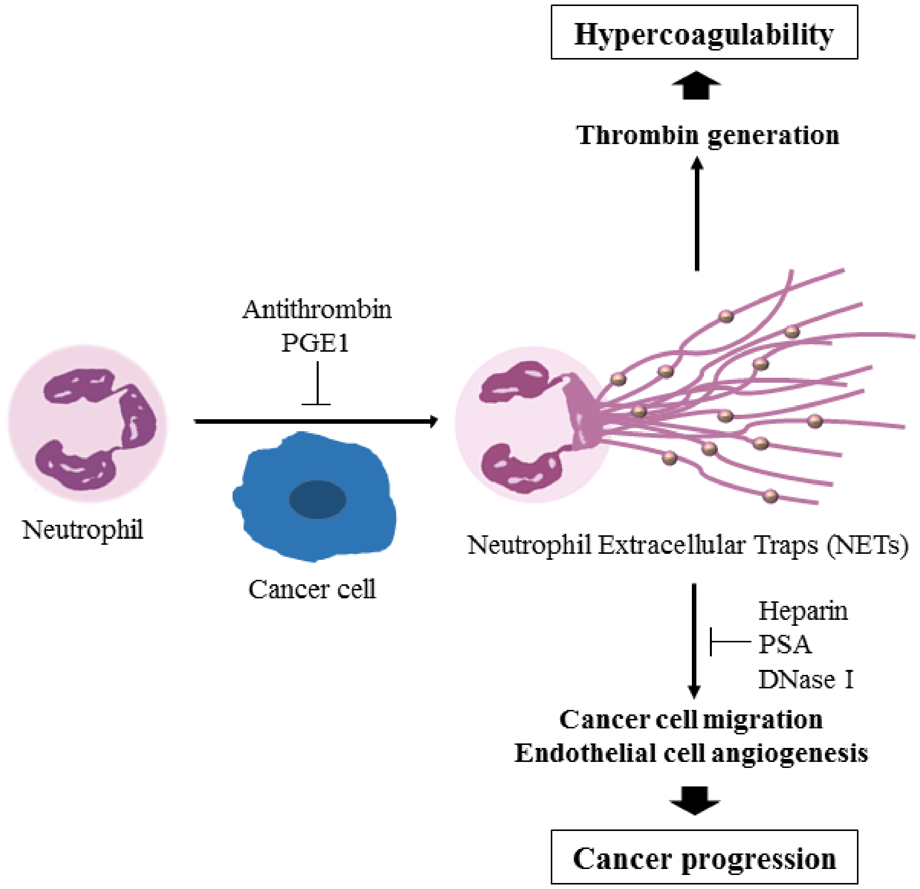

S4 Fig. Potential mechanism of how cancer cell–induced NET formation promotes both hypercoagulability and cancer progression. Cancer cells induce neutrophils to release NETs, which is inhibited by prostaglandin E1 (PGE1, cAMP inducer) and antithrombin. The released NETs promote hypercoagulability by enhancing plasma thrombin generation. They also promote cancer progression by enhancing cancer cell migration and endothelial cell angiogenesis, which are inhibited by heparin, polysialic acid (PSA), and DNase I.
